# Correlation between Adalimumab concentration, anti-drug antibody and clinical efficacy in rheumatoid arthritis and ankylosing spondylitis

**DOI:** 10.1186/s42358-026-00560-0

**Published:** 2026-06-22

**Authors:** Xile Wang, Tingting Wu, Xiaoxuan Xia, Qianxi Xing, Jingtian Shi, Shengqian Xu

**Affiliations:** 1 Department of Rheumatology & Immunology, The First Affiliated Hospital of Anhui Medical University, No. 218, Ji-xi Road, Hefei, Anhui 230022 China; 2 Department of Hematology & Rheumatology, Deyu Medical Ma’anshan General Hospital, No. 45, Hubei Road, Huashan District, Ma’anshan, Anhui 243000 China

**Keywords:** Adalimumab, Rheumatoid arthritis, Ankylosing spondylitis, Anti-drug antibody, Efficacy

## Abstract

**Objective:**

To detect anti-drug antibody (ADA), Neutralizing Antibody (NAb) and Adalimumab (ADL) concentration in Rheumatoid arthritis (RA) and Ankylosing spondylitis (AS) and investigate correlations with clinical efficacy.

**Methods:**

78 active RA patients, 59 active AS patients and 30 age, sex matched healthy individuals were recruited. The treatment course was 24 weeks with assessments every 12 weeks. ADL concentration, ADA and NAb were measured by enzyme-linked immunosorbent assay (ELISA). SPSS 26.0 was applied for statistical analysis.

**Results:**

The ADA incidence in AS group was higher than that in RA group after 24 weeks of treatment. The ADL concentration in AS/RA group with ADA was lower than that without ADA after 12/24 weeks of treatment. At 24 weeks of treatment: In RA group, the Remission-Low activity group’s ADL concentration was higher than that of the Moderate-Severe activity group, and the ADA positive rate was lower (χ²=4.481, *P* = 0.034). In AS group, the Remission-Low activity group’s ADL concentration was higher than that of the High-Extreme high activity group, and the ADA positive rate was lower (χ²=5.184, *P* = 0.023). Logistic regression analysis showed that ADA at 12/24 weeks and disease course were risk factors for Moderate-Severe activity status in RA group and High-Extreme high activity status in AS group. In contrast, log₁₀(ADL concentration) at 24 weeks was a protective factor in RA, and log₁₀(ADL concentration) at 12 and 24 weeks were protective factors in AS.

**Conclusion:**

The total incidence of ADA, which is almost entirely NAb in RA and AS patients at 24 weeks after ADL treatment, is approximately 20%. AS patients show higher ADA incidence than RA patients. ADA correlates with lower ADL concentration and is closely related to disease activity.

## Introduction

The application of biological agents in the treatment of autoimmune diseases such as Rheumatoid arthritis (RA) and Ankylosing spondylitis (AS), as well as the influence of their immunogenicity on clinical efficacy are currently hot research issues at home and abroad. According to axial spondyloarthritis (SpA) treatment recommendations by the Asia Pacific League of Associations for Rheumatology (APLAR) in 2018, TNF-α antagonists are strongly recommended for patients who fail to respond to treatment with two non-steroidal anti-inflammatory drugs (NSAIDs) [1]. The RA treatment recommendations released by European League Against Rheumatism (EULAR) in 2022 suggest that for RA patients with adverse prognostic factors, including the presence of autoantibodies, high disease activity, early erosion, or failure to respond to two Conventional Synthetic Disease-Modifying Anti-Rheumatic Drug (csDMARD) treatments, csDMARD combined with Biological Disease-Modifying Anti-Rheumatic Drug (bDMARD) treatment is recommended [2].

In this study, 78 patients with active RA and 59 patients with active AS were treated with Adalimumab (ADL) for 24 weeks. Their clinical and laboratory indicators were recorded in detail. The improvement of their disease condition and disease activity was evaluated before and after treatment. ADL concentration, anti-drug antibody (ADA) and neutralizing antibody (NAb) were measured at 12 and 24 weeks to explore their influence on clinical efficacy, which helped to better understand the immunogenicity of ADL and its application in autoimmune diseases. These findings provided necessary diagnostic and therapeutic insights for future clinical work. All enrolled participants demonstrated high disease activity before treatment and good compliance.

## Materials and methods

The study was approved by the Ethics Committee of the First Affiliated Hospital of Anhui Medical University (PJ2022-03-14), and all participants provided written informed consent prior to enrollment, in accordance with the Declaration of Helsinki.

### Object of study

#### Inclusion criteria for RA

A total of 78 patients with active RA who were treated in the Department of Rheumatology and Immunology of our hospital from September 2020 to September 2022 were selected, including 72 females and 6 males. Age ranged from 27 to 83 years, with a mean age of 50.64 ± 1.35 years. The height ranged from 145 to 188 cm with a mean height of 159.51 ± 0.80 cm. Their weight ranged from 37 to 83 kg, with a mean weight of 54.90 ± 0.89 kg. The Body Mass Index (BMI) ranged from 15.94 to 26.67 kg/m², with a mean BMI of 21.56 ± 0.29 kg/m². The diagnosis of RA was strictly based on the 1987 American College of Rheumatology (ACR) classification criteria for RA [3] and the 2010 ACR/EULAR classification criteria for RA [4]. Patients with a definite diagnosis met the criteria of the Disease Activity Score in 28 joints (DAS28) ≥ 3.2 [3–5]. Treatment status: The patients did not respond to regular csDMARD therapy for 12–24 weeks or could not tolerate the treatment.

#### Inclusion criteria for AS

A total of 59 patients with active AS who were treated in the Department of Rheumatology and Immunology of our hospital from September 2020 to September 2022 were selected, including 18 females and 41 males. Age ranged from 13 to 64 years, with a mean age of 33.27 ± 1.45 years. The height ranged from 153 to 183 cm with a mean height of 168.92 ± 0.96 cm. Their weight ranged from 45 to 91 kg, with a mean weight of 65.62 ± 1.59 kg. The BMI ranged from 16.33 to 29.40 kg/m², with a mean BMI of 22.89 ± 0.43 kg/m². The patients were diagnosed according to the 1984 New York classification criteria for AS [6]. Patients with a definite diagnosis met the criteria of the Ankylosing Spondylitis Disease Activity Score (ASDAS) ≥ 2.1 [7–9]. Treatment status: The patients did not respond to regular csDMARD therapy for 12 weeks or could not tolerate the treatment.

#### Inclusion criteria for healthy person

Thirty healthy individuals who underwent physical examination in the physical examination center of our hospital during the same period were selected, and their serum was collected to prepare positive quality controls. The positive quality control was prepared by spiking anti-Humira (Adalimumab) antibody into mixed healthy serum.

#### Exclusion criteria for all enrolled patients

1. Excluded patients with severe complications or a history of joint or spinal surgery within 4 weeks.
2. Excluded patients allergic to any component of the study drug.
3. Excluded patients who had used biological agents within 24 weeks.
4. Excluded patients with a history of tuberculosis or active tuberculosis, and those with a positive T-SPOT test.
5. Excluded patients with serious cardiovascular diseases, severe liver or kidney dysfunction, tumors and other chronic diseases, as well as acute and chronic infections.
6. Excluded patients with acquired immune deficiency syndrome (AIDS) or other serious infectious diseases.
7. Excluded pregnant or lactating patients.

#### Treatment regimen

The treatment regimen for RA was methotrexate (MTX) 10 mg every week orally, combined with ADL (BAT1406) 40 mg subcutaneously every two weeks. The treatment regimen for AS was celecoxib 0.4 g orally every night, combined with ADL 40 mg subcutaneously every two weeks. The ADL treatment course was 24 weeks, with an assessment every 12 weeks.

### Assessment of disease activity in RA patients

The disease activity indicators of each RA patient were recorded in detail, including DAS28, tender joint count (TJC), swollen joint count (SJC), joint function, morning stiffness duration, visual analogue scale (VAS), physician global assessment (PGA), health assessment questionnaire (HAQ) score, erythrocyte sedimentation rate (ESR), c-reactive protein (CRP), anti-cyclic citrullinated peptide (anti-CCP) antibody, rheumatoid factor (RF), etc.

DAS28 is a commonly used clinical index that can effectively evaluate the disease activity of RA patients. Its calculation formula is as follows [10]: DAS28 = 0.56×sqrt(TJC28) + 0.28×sqrt(SJC28) + 0.70×ln(ESR) + 0.014×PGA(PGA: VAS 0 ~ 10 cm). Its grouping criteria are as follows [11]: Low disease activity group (DAS28 < 3.2), Moderate disease activity group (3.2 ≤ DAS28≤5.1), Severe disease activity group (DAS28 > 5.1). According to the literature review, the standard treatment of RA targets clinical remission, that is, DAS28 ≤ 2.6. When the above criteria cannot be met, low disease activity can be used as a treatment indicator, that is, DAS28 ≤ 3.2 [5]. In our research, all RA patients treated for 24 weeks were divided into two groups according to DAS28 ≥ 3.2 (Moderate-Severe activity group) and DAS28 < 3.2 (Remission-Low activity group).

### Assessment of disease activity in AS patients

The disease activity indicators of each AS patient were recorded in detail, including ASDAScrp, Bath Ankylosing Spondylitis Disease Activity Index (BASDAI), Bath Ankylosing Spondylitis Functional Index (BASFI), PGA, morning stiffness duration, X-ray stage, joint function, occipital wall distance, thoracic dilation, digital ground distance, lumbar motion, ESR, CRP, etc.

ASDAS is an effective index commonly used in clinical practice to evaluate the disease activity of AS patients [7]. In this study, we used ASDAScrp for relevant statistical analysis. The ASDAScrp grouping criteria we adopted are as follows [8]: Inactivity/remission group (ASDAS < 1.3); Low disease activity group (1.3 ≤ ASDAS < 2.1); High disease activity group (2.1 ≤ ASDAS ≤ 3.5); Extremely high disease activity group (ASDAS > 3.5). All AS patients at 24 weeks of treatment were divided into two groups according to ASDAScrp ≥ 2.1(High-Extreme high activity group) and ASDAScrp < 2.1(Remission-Low activity group).

### Specimen collection and handling

In this study, blood samples from enrolled patients and healthy people were collected in the First Affiliated Hospital of Anhui Medical University, and all participants were fasting before blood collection. The collected peripheral blood was placed at room temperature (37℃) for 30 min before centrifugation, and the resulting serum was stored at -80℃ until use.

### Determination of ADL concentration, ADA, and NAb

ELISA was used to determine the concentration of ADL (BAT1406, batch number: 02A20201017), ADA and NAb in peripheral blood of each subject. For ADA, the screening cut-off value was 1.21 and the confirmation cut-off value was 36.8%, determined by normalizing signal values from repeated analysis of pre-dose blank serum samples (two operators, three days). The sensitivity was 3.125 ng/mL (regulatory requirement: ≤100 ng/mL). Drug tolerance was demonstrated at concentrations ≥ 50 ng/mL with 12.5 µg/mL ADL, and ≥ 20 ng/mL with 3.125 µg/mL ADL. For NAb, the cut-off value was 93.1% (same method), with a sensitivity of 125 ng/mL (regulatory requirement: ≤500 ng/mL), and drug tolerance was demonstrated at a concentration of ≥ 500 ng/mL with 12.5 µg/mL ADL.

### Data analysis

SPSS 26.0 statistical software was applied, and t-test, χ²-test, non-parametric test and multivariate Logistic regression analysis (Backward LR, likelihood ratio) were used to analyze all indicator data.

## Result

### The incidence of ADA and NAb were compared between AS and RA

After 12 weeks of treatment, the ADA incidence in AS patients was 31.3% (10/32), and all ADA was NAb. The ADA incidence in RA patients was 12.5% (5/40), and 80% of ADA was NAb. The difference in ADA incidence between the two groups was not statistically significant (χ²=3.789, *P* = 0.052). After 24 weeks of treatment, the ADA incidence in AS patients was 27.1% (16/59), and it was significantly higher than that in RA patients (9.0%, 7/78) (χ²=7.916, *P* = 0.005). All ADA was NAb in AS group and 85.7% was NAb in RA group. In the AS group, 8 patients had positive ADA at both 12 and 24 weeks, 2 patients had positive ADA at 12 weeks and negative ADA at 24 weeks, and 8 patients had negative ADA at 12 weeks and positive ADA at 24 weeks. In the RA group, 3 patients had positive ADA at both 12 weeks and 24 weeks, 2 patients had positive ADA at 12 weeks and negative ADA at 24 weeks, and 4 patients had negative ADA at 12 weeks and positive ADA at 24 weeks. Therefore, when 12 and 24 weeks of treatment were combined together, the ADA incidence was 30.5% (18/59) in AS group and 11.5% (9/78) in RA group. The difference was statistically significant (χ²=7.639, *P* = 0.006). The ADA incidence was 19.7% (27/137) when RA and AS were evaluated together at 12 and 24 weeks of treatment.

In the AS group, after 12 weeks of treatment, 3 patients with an ADL concentration of 0 produced ADA, all of which were NAb; the other 7 patients, who had low ADL concentration (< 800 ng/mL), also produced ADA. After 24 weeks of treatment, 12 patients with an ADL concentration of 0 produced ADA, all of which were NAb; the other 4 patients with low ADL concentration (< 900 ng/mL) produced ADA. In the RA group, after 12 weeks of treatment, among the 2 patients with an ADL concentration of 0, one produced ADA (which was NAb) and the other one did not produce ADA. The remaining 4 patients had low ADL concentration (< 600 ng/mL) and produced ADA. After 24 weeks of treatment, among the 6 patients with an ADL concentration of 0, 5 produced ADA (all NAb), and the other one produced non-neutralizing antibody (non-NAb). In addition, one patient who produced ADA had low ADL concentration (< 600 ng/mL).

### Comparison of ADL concentrations in AS and RA patients with or without ADA during each course of treatment

After 12 weeks and 24 weeks of treatment, ADL concentration in AS group with ADA was significantly lower than that without ADA (*P* < 0.0001). And the result was the same in RA group (*P* = 0.002 and *P* < 0.0001, respectively). (See Table 1)

**Table 1:** Comparison of ADL concentration between AS/RA patients with or without ADA

Group	ADL concentration (ng/mL)	ADA-negative group	ADA-positive group	Z	*P*
AS	12 weeks	8114.71(5785.34, 10301.13)	79.28(0, 162.71)	4.474	**< 0.0001**
	24 weeks	6288.44(3597.06, 9991.17)	0(0, 62.74)	5.821	**< 0.0001**
RA	12 weeks	7833.22(4863.25, 9721.51)	383.18(36.43, 3343.21)	3.026	**0.002**
	24 weeks	7152.92(3942.94, 10701.88)	0(0, 0)	4.293	**< 0.0001**

### Comparison of ADL concentration (at 24 weeks) and ADA positive rate (at 24 weeks) between RA patients with different disease activity groups after 24 weeks of treatment

All RA patients treated for 24 weeks were divided into two groups according to DAS28 ≥ 3.2(Moderate-Severe activity group) and DAS28 < 3.2(Remission-Low activity group) and the comparison between the two groups showed that: The ADL concentration in the Remission-Low activity group was significantly higher than that in the Moderate-Severe activity group (*P* = 0.003), and the ADA positive rate was obviously lower [2.2%(1/46) VS 18.8%(6/32), χ²=4.481, *P* = 0.034]. (See Table 2)

**Table 2:** Comparison of ADL concentration (at 24 weeks) and ADA positive rate (at 24 weeks) between RA patients with different disease activity after 24 weeks of treatment

Index	Moderate-Severe activity group (*N* = 32)	Remission-Low activity group (*N* = 46)	Z/χ²	*P*
ADL concentration at 24 weeks(ng/mL)	3793.98(561.32, 8594.95)	7746.82(4588.17, 11391.44)	2.962	**0.003**
ADA positive rate at 24 weeks (%)	6(18.8)	1(2.2)	4.481	**0.034**

### Comparison of disease-related indicators between RA patients with or without ADA after 24 weeks of treatment

At 24 weeks of treatment in RA patients, Morning stiffness duration, CRP and DAS28 in ADA-positive group were significantly higher than those in ADA-negative group(*P* < 0.05), and BMI was slightly lower (*P* < 0.05). (See Table 3)

**Table 3:** Comparison of disease-related indicators between RA patients with or without ADA after 24 weeks of treatment

Index	ADA-negative group *N* = 71	ADA-positive group *N* = 7	t/ Z/χ²	*P*
Disease course(year)	2.50(1.10, 11.00)	4.60(2.80, 9.03)	0.709	0.478
Age	51.05 ± 12.46	46.83 ± 8.77	0.480	0.633
Weight (kg)	53.50(50.00, 60.00)	52.50(50.00, 60.00)	0.167	0.867
Height (cm)	158.00(155.00, 161.00)	162.50(158.75, 167.25)	1.547	0.122
BMI (kg/m²)	21.56 ± 2.64	20.50 ± 1.24	2.344	**0.035**
swollen joint count	0(0, 1)	1(0, 3)	0.782	0.434
tender joint count	0(0, 2.00)	4.00(1.50, 12.00)	1.886	0.059
morning stiffness duration (min)	0(0, 5.00)	10.00(3.75, 37.50)	2.981	**0.003**
VAS score	2.00(1.00, 4.00)	2.00(0.75, 4.25)	0.214	0.831
HAQ score	0(0, 0.30)	0.05(0, 0.19)	0.480	0.631
anti-CCP antibody (RU/mL)	894.00(173.50, 1127.00)	950.50(447.25, 1198.25)	0.554	0.579
ESR (mm/h)	14.00(6.50, 28.00)	26.50(13.25, 47.00)	1.820	0.069
RF (IU/mL)	32.00(17.50, 72.95)	129.90(8.25, 413.55)	1.604	0.109
CRP (mg/L)	1.20(0.50, 3.60)	9.45(1.04, 18.20)	2.021	**0.043**
DAS28 score	2.76 ± 1.06	4.02 ± 0.94	2.714	**0.008**
ΔDAS28 score	2.29 ± 1.20	2.13 ± 1.46	0.123	0.902

### Comparison of ADL concentration (at 24 weeks) and ADA positive rate (at 24 weeks) between AS patients with different disease activity groups after 24 weeks of treatment

All AS patients treated for 24 weeks were divided into two groups according to ASDAScrp ≥ 2.1(High-Extreme high activity group) and ASDAScrp < 2.1(Remission-Low activity group). The comparison between the two groups suggested that: The ADL concentration in the Remission-Low activity group was clearly higher than that in the High-Extreme high activity group (*P* = 0.001), and the ADA positive rate was obviously lower [22.0%, (11/50) VS 71.4%, (5/7), χ²=5.184, *P* = 0.023]. (See Table 4)

**Table 4:** Comparison of ADL concentration (at 24 weeks) and ADA positive rate (at 24 weeks) in AS patients between different disease activity groups after 24 weeks of treatment

Index	High-Extreme high activity group (*N* = 7)	Remission-Low activity group (*N* = 50)	Z/χ²	*P*
ADL concentration at 24 weeks (ng/mL)	0(0, 83.65)	5387.78(1270.68, 8968.20)	3.224	**0.001**
ADA positive rate at 24 weeks (%)	5(71.4)	11(22.0)	5.184	**0.023**

### Comparison of disease-related indicators between AS patients with or without ADA after 24 weeks of treatment

At 24 weeks of treatment in AS patients, ASDAScrp score, BMI, ESR and CRP in ADA-positive group were higher than those in ADA-negative group(*P* < 0.05). (See Table 5)

**Table 5:** Comparison of disease-related indicators between AS patients with or without ADA after 24 weeks of treatment

Index	ADA-negative group *N* = 43	ADA-positive group *N* = 16	t/ Z/χ²	*P*
ASDAcrp score	1.13(0.66, 1.48)	1.86(1.03, 2.54)	2.469	**0.014**
ΔASDAScrp score	1.43 ± 1.16	0.85 ± 0.75	1.780	0.081
Age	33.40 ± 11.05	32.94 ± 11.82	0.139	0.890
Weight(kg)	63.78 ± 11.87	70.56 + 12.06	1.944	0.057
Height(cm)	169.00 ± 7.64	168.69 ± 6.78	0.144	0.886
BMI (kg/m²)	22.21 ± 3.06	24.71 ± 3.27	2.740	**0.008**
disease course (year)	4.50(1.95, 10.50)	3.50(1.00, 10.50)	1.136	0.256
joint function (I: II: III: IV)	38: 4: 1: 0	12: 4: 0: 0	2.737	0.254
X-ray staging (0: I: II: III: IV)	0: 0: 16: 22: 5	0: 0: 8: 8: 0	2.333	0.312
ESR (mm/h)	3.50(2.00, 11.00)	17.00(9.00, 25.00)	4.224	**< 0.0001**
CRP (mg/L)	1.27(0.58, 2.40)	4.00(3.00, 16.10)	3.172	**0.002**
occiput wall distance(cm)	0(0, 0.25)	0(0, 0)	0.218	0.827
thoracic dilation(cm)	4.00(2.75, 5.63)	5(5, 6)	1.722	0.085
lumbar motion(cm)	6(3, 10)	5(5, 10)	0.574	0.566
digital ground distance(cm)	14.00(4.50, 23.25)	10.00(0, 20.00)	0.833	0.405
BASDAI score	1.20(0.40, 2.60)	1.05(0.20, 2.10)	0.350	0.726
BASFI score	0.05(0, 1.00)	0(0, 0.60)	0.028	0.978
PGA score	2(1, 3)	1(1, 3)	0.081	0.935

### Logistic regression analysis of disease activity in RA and AS patients after ADL treatment

Defining DAS28 at 24 weeks of treatment as the dependent variable (0: DAS28 < 3.2, 1: DAS28 ≥ 3.2), and disease course, ADL concentration (24 weeks), ADL concentration (12 weeks), ADA (12/24 weeks,0 = negative,1 = positive), anti-CCP antibody, RF and HAQ were defined as the independent variables, and corrected for age, sex and BMI. Odds ratios for ADL concentration (OR = 1.000) are difficult to interpret due to the scale of the variable (ng/mL), so we have performed a log₁₀ transformation on the ADL concentrations at 12 and 24 weeks of treatment. The results showed that: In univariate analysis, ADA at 12/24 weeks (OR = 6.160, 95%CI 1.187–31.958, *P* = 0.030) and disease course (OR = 1.082, 95%CI 1.020–1.149, *P* = 0.009) were risk factors for Moderate-Severe disease activity status in RA patients. Log₁₀(ADL concentration) at 24 weeks was a protective factor for the same outcome (OR = 0.446, 95%CI 0.232–0.859, *P* = 0.016). After correction for age, sex and BMI, ADA at 12/24 weeks (OR = 10.272, 95%CI 1.621–65.097, *P* = 0.013) and disease course (OR = 1.072, 95%CI 1.005–1.143, *P* = 0.034) remained risk factors, and log₁₀(ADL concentration) remained protective (OR = 0.491, 95%CI 0.267–0.920, *P* = 0.022). (See Table 6)

Defining ASDAScrp at 24 weeks of treatment as the dependent variable (0: ASDAScrp < 2.1, 1: ASDAScrp ≥ 2.1), and disease course, ADL concentration (24 weeks), ADL concentration (12 weeks), ADA (12/24 weeks,0 = negative,1 = positive) were defined as independent variables, and corrected for age, sex and BMI. Since the odds ratios for ADL concentration were approximately 1.000, we similarly performed a log₁₀ transformation on the ADL concentrations at 12 and 24 weeks of treatment. The results indicated that: In univariate analysis, ADA at 12/24 weeks (OR = 19.000, 95%CI 2.075-173.937, *P* = 0.009) and disease course (OR = 1.132, 95%CI 1.008–1.271, *P* = 0.036) were risk factors for High-Extreme high disease activity status in AS patients. Log₁₀(ADL concentration) at 12 weeks (OR = 0.385, 95%CI 0.170–0.876, *P* = 0.023) and at 24 weeks (OR = 0.459, 95%CI 0.271–0.780, *P* = 0.004) were protective factors for the same outcome. After correction for age, sex and BMI, ADA at 12/24 weeks (OR = 19.000, 95%CI 2.075-173.937, *P* = 0.009) and disease course (OR = 1.132, 95%CI 1.008–1.271, *P* = 0.036) continued to be risk factors, whereas log₁₀(ADL concentration) at 12 weeks (OR = 0.385, 95%CI 0.170–0.876, *P* = 0.023) and at 24 weeks (OR = 0.459, 95%CI 0.271–0.780, *P* = 0.004) were still protective factors for High-Extreme high disease activity status in AS patients. (See Table 6)

**Table 6:** Logistic regression analysis of disease activity in RA/AS patients after ADL treatment

Group	Independent variables	Univariate analysis	*P*	Multifactor analysis*	*P*
		OR (95% CI)		OR (95% CI)	
RA	ADL concentration at 24 weeks	1.000(1.000–1.000)	0.007	1.000(1.000–1.000)	0.016
	ADL concentration at 12 weeks	1.000(1.000–1.000)	0.529	1.000(1.000–1.000)	0.351
	log₁₀(ADL concentration) at 24 weeks	0.446(0.232–0.859)	**0.016**	0.491(0.267–0.920)	**0.022**
	log₁₀(ADL concentration) at 12 weeks	1.242(0.602–2.563)	0.558	1.271(0.598–2.698)	0.533
	ADA at 12/24 weeks	6.160(1.187–31.958)	**0.030**	10.272(1.621–65.097)	**0.013**
	disease course	1.082(1.020–1.149)	**0.009**	1.072(1.005–1.143)	**0.034**
	HAQ	2.867(0.725–11.344)	0.133	1.853(0.422–8.128)	0.414
	anti-CCP antibody	1.001(1.000-1.001)	0.307	1.000(0.999–1.002)	0.398
	RF	1.002(0.999–1.006)	0.201	1.002(0.998–1.006)	0.293
AS	ADL concentration at 24 weeks	0.999(0.996–1.001)	0.153	0.999(0.996–1.001)	0.153
	ADL concentration at 12 weeks	0.996(0.984–1.007)	0.488	0.996(0.984–1.007)	0.488
	log₁₀(ADL concentration) at 24 weeks	0.459(0.271–0.780)	**0.004**	0.459(0.271–0.780)	**0.004**
	log₁₀(ADL concentration) at 12 weeks	0.385(0.170–0.876)	**0.023**	0.385(0.170–0.876)	0.023
	ADA at 12/24 weeks	19.000(2.075-173.937)	**0.009**	19.000(2.075-173.937)	**0.009**
	disease course	1.132(1.008–1.271)	**0.036**	1.132(1.008–1.271)	**0.036**

*: Corrected for age, sex and BMI

## Discussion

The pivotal study by van Schie et al. [12] demonstrated that over 90% of ADA against infliximab (IFX) and > 97% against ADL are neutralising, primarily by targeting the drugs’ TNF binding region (the paratope). This conclusion was further endorsed by Rinaudo-Gaujous et al. [13], who highlighted the critical clinical implication: the vast majority of ADA is neutralising. This immunodominance of the paratope provides a direct mechanistic explanation for our findings, linking ADA positivity to a significantly elevated risk of high disease activity. In our study, at 12 and 24 weeks of treatment, all ADA detected in the AS group was NAb. At 12 weeks of treatment, 80% of ADA in the RA group was NAb, and at 24 weeks, 85.7% was NAb. The results are similar to previous studies, which confirm that most ADA generated against ADL is NAb, and this has a major impact on the efficacy of drugs.

As for the incidence of ADA, in our study, it was 31.3% in AS patients and 12.5% in RA patients after 12 weeks of treatment, and the results were 27.1% and 9.0%, respectively, after 24 weeks of treatment. For the whole course of treatment, the ADA incidence was 30.5% in AS group and 11.5% in RA group. Meanwhile, when RA and AS patients were combined, the result was 19.7%. Comparatively, the findings of this study align closely with those of previous research. Eva L. Kneepkens et al. [14] found that 27% of AS patients developed ADA after 24 weeks of treatment. Ariela Hoxha et al. [15] found that the ADA incidence was 19% in inflammatory arthritis patients treated with ADL, and 90.9% of ADA positivity appeared within the 4th week of treatment. In a study of 39 patients with axial SpA treated with TNF-α antagonist, 15% of the patients developed ADA, and the study showed that ADA-positive patients had significantly higher disease activity than ADA-negative patients [16]. Another study reported that the ADA incidence was 29.0% in active inflammatory arthritis treated with ADL [17]. This suggests that when ADL is used to treat RA and AS, approximately one-fifth of patients will develop ADA, especially in the early stage of treatment. The determination of ADA at early and steady-state levels using appropriate methods can contribute to the evaluation of immunogenicity for ADL.

We also found that the incidence of ADA in the AS group was significantly higher than that in the RA group at 24 weeks of treatment (χ²=7.916, *P* = 0.005). This difference may be attributed to the different treatment regimens between the two groups. Studies showed that MTX could reduce the immunogenicity of ADL in RA patients in a dose-dependent manner, delay the generation of ADA or reduce the detection rate of ADA [18, 19]. The ADL concentration correspondingly increased with the increasing MTX dosage and the recommended dose of MTX is 10 mg orally once a week. A study also suggested that the generation of ADA is inevitable, and the combination of MTX or leflunomide can effectively prevent the generation of ADA [20]. In a study of RA patients receiving IFX therapy, MTX was associated with a lower risk of developing anti-infliximab antibodies (ATI) and a reduction in TNF-α bioactivity compared with other csDMARDs. These findings suggest that MTX plays an important role in modulating TNF bioactivity and inhibiting the production of antibodies against IFX [21]. Consequently, in ADL treatment, combining MTX or other immunosuppressants can reduce ADA production and its adverse effects.

This study also found that after 12 and 24 weeks of treatment, the ADL concentration in AS patients with positive ADA was significantly lower than that in patients with negative ADA(*P* < 0.0001), and this pattern was also found in RA patients (*P* = 0.002 and*P* < 0.0001, respectively). In addition, in both AS and RA patients, regardless of whether it was at 12 or 24 weeks, most of the ADA produced was NAb, and the ADL concentration of the corresponding patients was 0 or low. These results indicate that ADA and ADL immunogenicity are closely related. Due to the influence of ADL immunogenicity, the production of ADA, particularly NAb, leads to different degrees of reduction in ADL concentration. Similar findings have been found in previous studies. Geertje M Bartelds et al. [22] found that during the treatment of RA, ADL concentration in the ADA-positive group was significantly lower than that in the ADA-negative group. Eva L Kneepkens et al. [14] also found this result in AS patients.

Clinical studies have shown that ADA production significantly reduces drug efficacy of ADL therapy, often leads to diminished patient response, poor treatment outcomes, or even failure. This mainly involves drug clearance in the body. It is known that through the formation of immune complexes, ADA can neutralize or alter the clearance of biological agents and affect the metabolism of drugs. This can directly reduce effective drug concentrations in patients and lead to diminished efficacy or varying degrees of adverse reactions [23, 24]. According to the study of M K de Vries et al. [25], after 24 weeks of ADL treatment in AS patients, ADA positive rate was significantly higher than ADA negative rate in non-responders (*P* = 0.012). Geertje M Bartelds et al. [26] also found that RA patients with positive ADA were more likely to suffer treatment failure, and the generation of ADA was associated with lower ADL concentration, lower disease activity or lower clinical remission possibility. Erik H Vogelzang et al. [27] found that Psoriatic arthritis patients with positive ADA showed lower ADL levels and worse outcomes than ADA-negative ones. In summary, during ADL treatment, ADA-positive patients generally have lower effective drug concentration and poorer drug efficacy compared with ADA-negative patients, and they are more prone to treatment failure.

In this study, after 24 weeks of treatment in both RA and AS groups, the ADL concentration (at 24 weeks) in the low activity group was significantly higher than that in the high activity group, and the ADA positive rate (at 24 weeks) was obviously lower. These results indicate that clinical response and disease activity are closely related to ADL concentration and ADA level in both RA and AS. Low ADL concentration and high ADA level are associated with high disease activity, which may lead to treatment failure. Our results are similar to those of previous studies to varying degrees.

We also found that at 24 weeks of treatment in RA patients, morning stiffness duration, CRP and DAS28 in the ADA-positive group were significantly higher than those in the ADA-negative group (*P* < 0.05), and BMI was slightly lower (*P* < 0.05). In AS patients, ASDAScrp score, BMI, ESR and CRP in the ADA-positive group were also higher (*P* < 0.05). These results also suggested that higher disease activity is generally associated with positive ADA during ADL therapy in RA and AS. One study indicated that higher baseline body fat content was independently associated with clinical response to TNF-α inhibitors in AS patients, and the higher the baseline fat content, the lower the possibility of clinical improvement after treatment [28]. In the AS group in this study, BMI in the ADA-positive group was higher than that in the ADA-negative group, and AS patients with higher BMI might be more prone to ADA formation, which might result in a lower response rate to ADL, further leading to poor clinical efficacy and higher disease activity. However, the opposite result was observed in the RA group, that is, the BMI of the ADA-positive group was slightly lower than that of the ADA-negative group (*P* < 0.05). Similar results have not been found in previous studies, so it is uncertain whether this result is universal. We acknowledge that the small sample sizes in certain subgroups of our study may limit our ability to perform more accurate analyses and interpretations regarding BMI. Further studies are warranted to validate and explain the impact of BMI on ADA development.

The logistic regression analysis identified ADA at 12/24 weeks and disease course as risk factors for Moderate-Severe activity status in RA and High-Extreme high activity status in AS. The odds of Moderate-Severe activity status in RA patients were 10.272 times higher in the ADA-positive group than in the ADA-negative group, while the odds of High-Extreme high activity status in AS patients were 19.000 times higher in the ADA-positive group than in the ADA-negative group. These results suggest that ADA markedly reduces the clinical response of RA and AS patients to ADL, predisposing them to high disease activity and leading to treatment failure. In contrast, log₁₀(ADL concentration) at 24 weeks was a protective factor in RA, and log₁₀ (ADL concentration) at 12 and 24 weeks were protective factors in AS. This further indicates that ADL concentration is an important factor affecting clinical efficacy, and higher ADL concentration is strongly associated with a favorable clinical response.

According to related reports, during the treatment of TNF-α inhibitors, the risk of adverse drug reactions is obviously enhanced in ADA-positive groups. It is known that ADA can bind to TNF-α inhibitors to form immune complexes, and this process may lead to acute infusion reactions of the drugs [29–31], which commonly include fever, headache, nausea, etc. It reminds us to be vigilant about these adverse reactions during the treatment.

However, one limitation of this study is the small size of the ADA-positive subgroup, which restricted our ability to perform more complex multivariable modeling that could have provided a more detailed understanding of the relationships between variables. Therefore, our findings should be considered exploratory, and larger prospective studies are needed to validate these results.

## Conclusion

This study demonstrated that the total incidence of ADA, which was predominantly NAb, reached approximately 20% at 24 weeks following ADL treatment in patients with RA and AS. The incidence of ADA was significantly higher in AS patients compared to that of RA. Importantly, the presence of ADA was correlated with lower ADL concentration and was associated with higher disease activity. Collectively, these findings provide robust clinical evidence supporting the efficacy and safety profile of ADL in the management of RA and AS. Furthermore, they highlight the potential utility of monitoring ADA and drug levels for the early identification of treatment failure, thereby facilitating timely and individualized therapeutic interventions. Larger prospective studies are needed to validate these subgroup findings.

## Data Availability

The datasets are available from the corresponding author upon reasonable request.

## Abbreviations

ACR: American College of Rheumatology
ADA: Anti-drug antibody
ADL: Adalimumab
AS: Ankylosing spondylitis
ASDAS: Ankylosing Spondylitis Disease Activity Score
BASDAI: Bath Ankylosing Spondylitis Disease Activity Index
BASFI: Bath Ankylosing Spondylitis Functional Index
BMI: Body Mass Index
anti-CCP: Anti-cyclic citrullinated peptide
CRP: C-reactive protein
DAS28: Disease Activity Score in 28 joints
bDMARD: Biological Disease-Modifying Anti-Rheumatic Drug
csDMARD: Conventional Synthetic Disease-Modifying Anti-Rheumatic Drug
ELISA: Enzyme-linked immunosorbent assay
ESR: Erythrocyte sedimentation rate
EULAR: European League Against Rheumatism
HAQ: Health assessment questionnaire
IFX: Infliximab
MTX: Methotrexate
NAb: Neutralizing Antibody
NSAIDs: Non-steroidal anti-inflammatory drugs
PGA: Physician global assessment
RA: Rheumatoid arthritis
RF: Rheumatoid factor
SJC: Swollen joint count
SpA: Spondyloarthritis
Tab: Table
TJC: Tender joint count
VAS: Visual analogue scale
